# Potential angiotensin converting enzyme (ACE) inhibitors from Iranian traditional plants described by Avicenna’s Canon of Medicine

**Published:** 2019

**Authors:** Seyede Zohre Kamrani Rad, Behjat Javadi, A.Wallace Hayes, Gholamreza KarimI

**Affiliations:** 1 *Department of Pharmacodynamics and Toxicology, School of Pharmacy, Mashhad University of Medical Sciences, Mashhad, Iran.*; 2 *Department of Traditional Pharmacy, School of Pharmacy, Mashhad University of Medical Sciences, Mashhad, Iran.*; 3 *University of South Florida College of Public Health, Tampa, FL USA and Michigan State University Institute for Integrative Toxicology, East Learning; MI USA.*; 4 *Pharmaceutical Research Center, Institute of Pharmaceutical Technology, Mashhad University of Medical Sciences, Mashhad, Iran.*

**Keywords:** Hypertension, Angiotensin converting enzyme, Avicenna, Canon of medicine, Traditional plants

## Abstract

**Objective:**

Hypertension is an important cause of cardiovascular disorders. The angiotensin converting enzyme (ACE) plays an important role in hypertension; therefore, inhibition of ACE in treatment of chronically elevated blood pressure is an important therapeutic approach. In the current review, we have provided information from Persian Traditional Plants described by Avicenna in the Canon of Medicine and a number of more current scientific databases, with a focus on angiotensin converting enzyme inhibitory activity of the following six plants: *Allium sativum, Cinnamomum zeylanicum, Jasminum grandiflorum, Tribulus terrestris, Vaccinium myrtillus *and *Vitis vinifera*.

**Materials and Methods:**

A literature search was conducted and information on different traditional plants used for hypertension was collected from the Canon of Medicine and several other databases including PubMed, Scopus, Google Scholar and Web of Science.

**Results:**

The present article highlights the antihypertensive potential of the above-noted six plants*. *Administered doses, manner of consumption, types of extracts, preparations and derivatives, personal habits, and other geographic and epidemiologic variables have an important role in the potential efficacy of these plants.

**Conclusion:**

Recent studies indicated a significant correlation between the traditional use of Persian plants to reduce blood pressure and angiotensin converting enzyme inhibitory activity.

## Introduction

**Figure 1:**
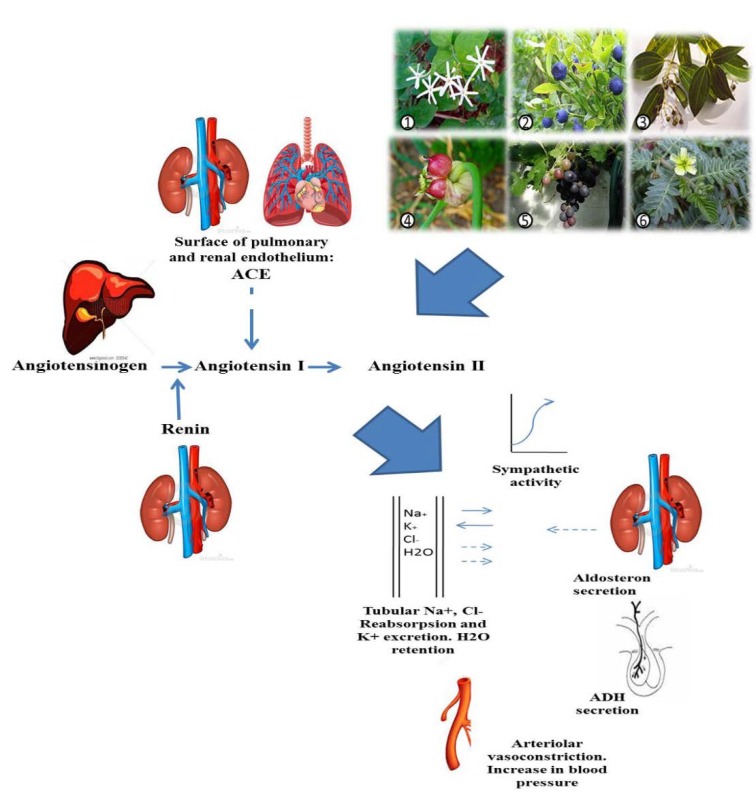
Schematic diagram showing the proposed ACE – inhibitory activity of six Iranian traditional plants

The eighth report of the Joint National Committee on prevention, detection, evaluation and treatment of high blood pressure categorized hypertension into three stages comprising pre-hypertension, and stages 1 and 2;a systolic blood pressure (BP) of 120-139mmHg or a diastolic BP of 80-89mmHg defined as pre-hypertension (Dennison-Himmelfarb et al., 2013 ▶).Hypertension affects about a billion people of the world population at some time-points during their lifetime and its prevalence increases with age(Emtiazy et al., 2014 ▶). One of every two persons over 65 years of age develop high BP (Chobanian et al., 2003 ▶). The angiotensin converting enzyme (ACE) plays an important role in hypertension via enhancing vasoconstriction and maintenance of peripheral resistance; therefore, inhibition of ACE is widely recommended as a therapeutic target for treatment of high BP (Barbosa-Filho et al., 2006 ▶) (Figure 1). 

1. *Jasminum grandiflorum*, 2. *Vaccinium myrtillus*, 3. *Cinnamomum zeylanicum*, 4. *Allium sativum*, 5. *Vitis vinifera*, and 6. *Tribulus terrestris.*

Avicenna was a famous Persian physician and one of the leading scientists of his time (Moosavi, 2009 ▶). In his well-known book ‘Canon of Medicine’, he dedicated a chapter to ‘Emtela’ which means ‘repletion’ or ‘plethora’. In this chapter, he described etiology, features and complications of emtela which is well-matched to high BP, although hypertension was not considered a disease in Persian medical textbooks (Emtiazy et al., 2014 ▶; Kardeh et al., 2014 ▶). Moreover, other records of Traditional Persian Medicine (TPM) have also mentioned emtela and its signs, symptoms and treatment(Jorjani, 1976 ▶).

Numerous adverse reactions related to the antihypertensive drugs may limit their usage and subsequently reduce treatment adherence. Therefore, developing new medications with limited adverse effects and higher efficacies is a research focus for the treatment of cardiovascular disorders (CVD).

**Figure 2:**
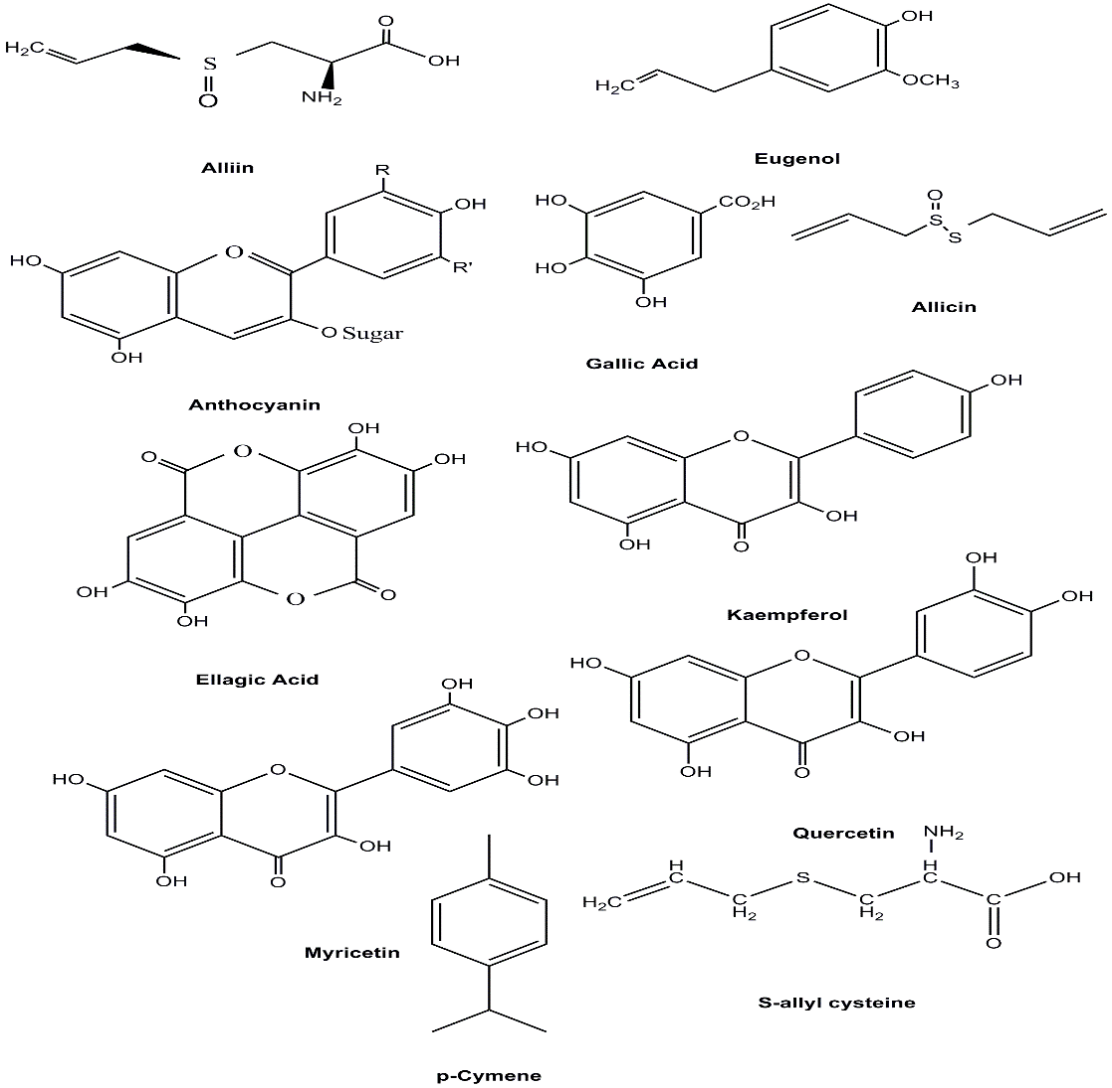
Chemical structures of some active components of Traditional Persian Medicine Plants

There are several researches on the anti-hypertensive properties of plants (Somanadhan et al., 1998 ▶; Somanadhan et al., 1999 ▶). Also, a number of reports showed the ACE inhibitory [ACEI] activity of medicinal plants (Meunier et al., 1987 ▶; Khan et al., 2001 ▶; Loizzo et al., 2008 ▶; Patten et al., 2016 ▶). Plants with some ACE inhibitor activity are listed in table 2 and IC50 or % inhibition (mg/ml) is shown in table 1. In Iran, about 40 plant species are used to treat hypertension (Baharvand-Ahmadi and Asadi-Samani, 2016 ▶). Plants with some ACE inhibitory activity are listed in Table 2. Among these, six were reported by TPM as ACE inhibitors and are currently used in Iran to treat BP. The chemical structures of some active components of these plants are shown in Figure 2. 

The present study reports the anti-ACE activity of six medicinal plants recommended by TPM. In recent years, some of the plants suggested by Avicenna have been the subject of pharmacological and clinical trials. Herein, we searched

scientific databases to verify the effectiveness of the medicinal plants suggested by Avicenna for the treatment of hypertension. Moreover, major bioactive compounds of these plants are also discussed.

**Table 1:** ACE inhibitor activity of Traditional Persian Medicine Plants

**Plant name**	**IC50 or % inhibition (mg/ml)**	**Part of plant**	**References**
*Allium sativum*	58% at 0.3	Bulb	(Sendl et al., 1992 ▶)
*Cinnamomum zeylanicum*	87% at 0.2	Bark	(Inokuchi et al., 1984 ▶)
*Jasminum grandiflorum*	78% at 0.33	Aerial	(Somanadhan et al., 1999 ▶)
*Tribulus terrestris*	50% at 0.33	Aerial	(Somanadhan et al., 1999 ▶)
*Vaccinium myrtillus*	0.0025	Leaf	(Persson et al., 2009 ▶)
*Vitis vinifera*	0.08	Fruit	(Meunier et al., 1987 ▶)

## Materials and Methods

 A literature search was conducted and information on the effect of six traditional plants used against hypertension, was collected from the Canon of Medicine and several other databases including PubMed, Scopus, Google Scholar and Web of Science using the following keyword: Iranian traditional plants, hypertension, ACE, Canon of medicine, Avicenna, *Allium sativum, Jasminum grandiflorum, Cinnamomum zeylanicum, Tribulus terrestris, Vaccinium myrtillus *and *Vitis vinifera*. No time restriction was considered for selection of published studies, in this review.

## Results

***Allium sativum*** **L.**

**Plant description and distribution**

*Allium sativum, *commonly known as garlic is a member of Alliaceae family (Patten et al., 2016 ▶). Garlic is a bulbous plant that is cultivated all around the world, but it originally was native to central and southern Asia (Elkayam et al., 2001 ▶; Rastogi et al., 2016 ▶). Various parts of the plant including green garlic leaves, seeds, stalks, flowers and bulbs are traditionally used as food and medicine. Different preparations of garlic such as oil, macerate, powder, aged garlic, allicin powder extract, etc. are used (Arzanlou and Bohlooli, 2010 ▶). 

**History of use**

In different cultures, garlic has been used as a spice, food additive and as herbal medicine (Elkayam et al., 2001 ▶). The records of garlic consumption date back to 5000 years ago (Rastogi et al., 2016). It has been used for treatment of several ailments such as headache, tumors, and intestinal worms (Corzo-Martínez et al., 2007 ▶). Garlic has been tried as a complementary treatment for heart disorders and insect bites (Rastogi et al., 2016 ▶). In Sanskrit and TPM literature, garlic was reported as a beneficial remedy for the treatment of chronic cough, toothache, constipation and septic diseases (Rastogi et al., 2016 ▶). Moreover, the Zoroastrian holy record, dating back to the sixth century BC., mentioned culinary and medicinal properties of garlic (Bayan et al., 2014 ▶). In “The Canon of Medicine’’ oral use and topical application of garlic has been reported to possess analgesic and anti-inflammation properties (Mahdizadeh et al., 2015 ▶).In addition, several reports suggested aged garlic extract as an important remedy for heart diseases and arterial obstructive disorders (Aviello et al., 2009 ▶; Mahdizadeh et al., 2015 ▶).

**Chemistry**

Chemical studies on garlic showed the presence of several enzymes, amino acid, organosulfur compounds such as S-allyl cysteine, alliin, allicin, γ-glutamyl cysteine and ajoenein in various parts of the plant (Sendl et al., 1992 ▶; McRae, 2006 ▶; Rastogi et al., 2016 ▶). Powdered garlic contains about 1% alliin which is metabolized by the enzyme, alliinase, to allicin. This process occurs when its bulb is crushed or cut. Garlic oil and aged garlic contain different products of allicin transformation (Rastogi et al., 2016 ▶). 

**Pharmacology**

Modern pharmacological studies confirmed that garlic has therapeutically useful properties against cardiovascular disorders, , chronic diseases related to aging and possibly cancer and for cancer prevention (Rastogi et al., 2016 ▶), as well as reducing blood glucose and inhibiting platelet aggregation (Elkayam et al., 2001 ▶). Other studies reported that S-allyl cysteine has anti-hepatotoxic and anti-oxidant effects and may reduce the incidence of stroke (Asdaq and Inamdar, 2010 ▶). According to McRae, allicin is responsible for inhibition of cholesterol synthesis (McRae, 2006 ▶). A number of pharmacological and clinical studies reported the anti-hypertensive activity of garlic and its major compounds (Duda et al., 2007 ▶; Ried et al., 2013 ▶; Ried et al., 2016 ▶).

Epidemiological studies demonstrated a significant correlation between garlic consumption and the reduction of systolic and diastolic blood pressure (Xiong et al., 2015 ▶). A meta-analysis conducted by Xiong and colleagues, demonstrated a correlation between garlic consumption and reduction of systolic and diastolic blood pressure. In these trials, patients with SBP≥140mmHg and/or DBP≥90mmHg received six garlic preparations including a dried garlic homogenate (188 mg), processed garlic capsules, garlic powder, aged garlic extract (960 mg), regular garlic pills (900 mg) and time-released garlic powder tablets (600 or 2400 mg/day). Mean systolic blood pressure (SBP) and diastolic blood pressure (DBP) were decreased by all of the garlic preparations indicating that garlic is an effective approach for lowering BP (Xiong et al., 2015 ▶).In a clinical trial conducted by McMahon and his colleagues, it was found that BP was significantly reduced5-14 h after consumption of 2400mg garlic tablets containing 31.2mg allicin in nine patients with severe hypertension (DBP≥115mmHg)(McMahon and Vargas, 1993 ▶). Another study carried out by Reid et al. in 50 patients with uncontrolled hypertension (SBP≥140 mmHg) showed that following consumption of aged garlic capsules (960 mg/day containing 2.4 mg S-allyl cystein) for 12 weeks, mean SBP was dramatically decreased (Ried et al., 2010 ▶). Controversially, Capraz et al.reported no significant BP-lowering effect for consumption of garlic cloves or garlic tablets (Cirkulin^®^) compared to control group (Capraz et al., 2007 ▶). These discrepancies may be attributed to differences in administered doses of garlic tablets, number of participants, personal habits and other geographic and epidemiologic variables.

A majority of reports, however, suggest that blood pressure lowering effect of garlic is attributed to its vasorelaxant activity and is mediated by release of endothelium-derived relaxing factor (EDRF) or muscle-derived relaxing factor (MDRF). Ozturk and his colleagues compared the relaxant effects of garlic and acetylcholine on the rat aorta, *in vitro*. The results demonstrated that garlic has a dose-dependent relaxant effect which is attenuated in the absence of endothelium but is not completely abolished. Therefore, it is speculated that EDRF may be responsible for the relaxant effects of garlic on the arterial smooth muscle tone (Öztürk et al., 1994 ▶).

Various reports suggested different mechanisms for garlic BP lowering effects including production of hydrogen sulfide (H_2_S), stimulation of nitric oxide (NO) (Ried et al., 2013 ▶;Shouk et al., 2014 ▶), inhibition of ACE (Hosseini et al., 2007 ▶; Oboh et al., 2013 ▶; Ried et al., 2013 ▶; Shouk et al., 2014 ▶; Jain et al., 2015 ▶) and blockage of α adrenergic receptors and calcium channels (Shouk, et al., 2014 ▶). Reid et al. (2014) ▶ reported that allicin has a low sustained bioavailability in human tissues, so its activity via inhibition of angiotensin-II production and vasodilation effects are less plausible than its NO- and H_2_S-mediated mechanisms (Ried and Fakler, 2014 ▶).

Animal experiments showed that administration of S-allyl cysteine and captopril can synergistically reduce BP via inhibition of ACE (Shouk et al., 2014 ▶). Sharifi et al also demonstrated ACEI effects of allicin in reduction of blood pressure (Sharifi et al., 2003 ▶). Oboh et al. (2013) ▶ studied the effect of phenolic extract of garlic on BP and reported that it can strongly act as an inhibitor of ACE, *in vitro*. In this study, evaluation of the free and bound phenolic inhibitory effects on ACE revealed that bound phenolics have more potent effect than the free phenolics in reduction of ACE activity; however, both inhibited malondialdehyde production (Asdaq and Inamdar, 2010 ▶) in a dose-dependent manner (Oboh et al., 2013 ▶).

***Cinnamomum zeylanicum*** **Blume**

**Plant description and distribution**

*Cinnamomum zeylanicum*, commonly known as cinnamon tree, is a member of the Lauraceae family (Patten et al., 2016 ▶). The genus *Cinnamomum* has about 250 species, 20 of which grow in India (Jayaprakasha and Rao, 2011 ▶). Various parts of the cinnamon tree including the bark, leaves, flowers, fruits and roots, are used as medicine or food additive (Ranasinghe et al., 2013 ▶).

**History of use**

cinnamon has been considered a medicinal plant in different countries and also has been widely exploited as a common spice for thousands of years (Jayaprakasha and Rao, 2011 ▶; Ranasinghe et al., 2013 ▶). Cinnamon oil is extensively used in flavors and foods as well as beverages, perfumery and pharmaceutical industries as a preservative (Jayaprakasha and Rao, 2011 ▶; Saleem et al., 2015 ▶). *C. zeylanicum *is largely found in tropical Asia and Sri Lanka. The fruits grow from May to August (Jayaprakasha and Rao, 2011 ▶). As a folk remedy, *C. zeylanicum *has been considered for the treatment of digestive, respiratory and gynecological ailments (Ranasinghe et al., 2013 ▶)and nervous stress(Malik et al., 2015 ▶).

**Chemistry**

Analysis of the volatile oil from the leaves of *C. zeylanicum* showed the presence of p-cymene and eugenol as the main components. Additional studies reported the presence of α-pinene, lomonen, cinnamaldehyde, copaene, β-cadinene, δ-cadinene, calamenene, 3,7 (II)-salinadiene, amorphene and O-methoxy cinnamaldehyde in *C. zeylanicum *essential oil (Saleem et al., 2015 ▶). Moreover, cinnzeylanine and cinnzeylanol were isolated from the dried bark of *C. zeylanicum *(Jayaprakasha and Rao, 2011 ▶).

**Table 2:** Summary of Iranian plants with ACE inhibitory activities

**Plant name** **(common name)**	**Family**	**Main isolated** **phytochemical constituents**	**Part of plant**	**Action**	**References**
*Allium sativum *L. (garlic)	Alliaceae	S-allyl cysteine, alliin, allicin, γ-glutamyl cysteine and ajoene		Cardioprotection Anti-oxidant Anti-hyperlipidemic Anti-hypertensive	(Asdaq and Inamdar, 2010 ▶; Rastogi et al., 2016 ▶) (McRae, 2006 ▶; Duda et al., 2007 ▶) (Ried and Fakler, 2014 ▶)
*Cinnamomum zeylanicum *Blume (true cinnamon or Ceylon)	Lauraceae	P-cymene,eugenol, cinnzeylanine and cinnzeylanol	Bark	Anti-oxidant	(Jain, et al., 2015 ▶) (Sultana et al., 2016 ▶) (Patten et al., 2016 ▶)
			“	Cough suppressant	
			“	Anti-hypertensive	
*Jasminum grandiflorum *L. (jasmine)	Oleaceae	Secoiridoid, iridoid glycosides, saponins, terpenoids and flavonoids	Leaf	Dermal ulcer healing	(Chaturvedi et al., 2013 ▶) (Patten et al., 2016 ▶) (Chaturvedi and Tripathi, 2011 ▶; Arun et al., 2016 ▶) (Venkataiah et al., 2013 ▶)
			Aerial	Anti-hypertensive	
			Leaf “ “	Anti-inflammation Anti-oxidant nephroprotective	
*Tribulus terrestris *L. (Gokhshura or puncture vine)	Zygophyllaceae	Furostanol, spirostanol saponins, sulphated saponins of tigogenin and diosgenin	Fruit	Nephroprotective	(Kavitha and Jagadeesan, 2006 ▶) (Tuncer et al., 2009 ▶) (Phillips et al., 2006 ▶)
				Anti-hyperlipidemic	
			Whole plant	Anti-hypertensive	
*Vaccinium myrtillus *L. (bilberry)	Ericaceae	Different anthocyanins such as galactosides and glycosides of peonidin, petunidin, delphinidin, malvidin and cyanidin		Neuroprotactive	(Matsunagaet al., 2009 ▶) (Lee et al., 2013 ▶)
			Fruit	Anti-oxidant Anti-hypertensive	
*Vitis vinifera *L. (grapevine)	Vitaceae	Phenolic components (myricetin, ellagic acid, kaempferol, gallic acid and quercetin), polyphenols (flavonoids, anthocyanins)	Seed	Radical scavenger	(Fauconneau et al., 1997 ▶; Facino et al., 1999 ▶; Aldini et al., 2003 ▶)
			“	Cardioprotective	
			Fruit	Anti-oxidant	
			Seed Stem bark	Anti-hypertensive Alzheimer’s disease	(Koo et al., 2008 ▶; Godse et al., 2010 ▶; Quiñones et al., 2013 ▶)
			Seed	Vasorelaxant	(Gharib Naseri et al., 2010 ▶)
			“	Cardioprotective	(Afonso et al., 2013 ▶)
			Seed Extract	Hypolipidemic	(Adisakwattana et al., 2010 ▶, Agrawal et al., 2010 ▶; Afonso et al., 2013 ▶)
			Leaf Extract	Bronchodilator	(Gharib Naseri and Heidari, 2006 ▶)

**Pharmacology**

Modern pharmacological reports suggested that *C. zeylanicum *improves cognitive impairment and oxidative stress (Jain et al., 2015 ▶), prevents carbon tetrachloride-induced damages on the male reproductive system (Yüce et al., 2014 ▶) and ameliorates inflammation and arthritis (for polyphenolic fraction of the bark)(Rathi et al., 2013 ▶).

*C. zeylanicum *was reported to have useful effects on blood pressure following short-term administration to patients with diabetes (Akilen et al., 2013 ▶) An extract of the bark was suggested as a potential anti-hypertensive agent (Kesari et al., 2014 ▶). It was also reported that a methanol extract of *C. zeylanicum *stem bark has both acute and chronic anti-hypertensive potential. At concentrations of 5, 10 and 20mg/kg, cinnamon was reported to reduce the mean arterial blood pressure (MABP) 12.5, 26.6 and 30.6%, respectively (Nyadjeu et al., 2013 ▶). 

A methanol extract of *C. zeylanicum *reduced the plasma level of triglycerides (TG) and total cholesterol up to 38.1% and 32.1%, respectively (also, decreases LDL-cholesterol (75.3%) and increases HDL-cholesterol (58.4%) in rats) (Nyadjeu, et al. 2013 ▶). A similar extract of dried bark also inhibited ACE in experimental animals (Barbosa-Filho et al., 2006 ▶). The anti-hypertensive mechanism was speculated to be mediated hrough elevation of endothelial NO and activation of the K-ATP channel in vascular smooth muscle (Nyadjeu et al., 2011 ▶).

In 2016, Ranjini and his colleagues determined the inhibitory effects of methanolic extract of *C. zeylanicum *on ACE activity in sheep tissues. In the presence of the extract, tissue ACE activity was reduced and these effects were more significant in the kidney than in the testis and lung tissues (Ranjini et al., 2016 ▶).

***Jasminum grandiflorum*** **L.**

**Plant description and distribution**

*Jasminum grandiflorum*, commonly known as jasmine is a member of the Oleaceae family (Sadhu et al., 2007 ▶; Sandeep, 2009 ▶; Ferreres et al., 2014 ▶). *Jasminum *is a genus of shrubs and vines containingaround200 species found all over the world (Arun et al., 2016 ▶). *J. grandiflorum *is domestic to temperate and tropical areas including parts of Asia, Kashmir, Philippines, Myanmar and Sri Lanka (Sandeep, 2009 ▶; Arun et al., 2016 ▶). It is distributed across the west coast of India from south Canara to the low elevations of Kerala and is also cultivated in Italy, China, India, France, Egypt and Morocco (Somanadhan et al., 1998 ▶; Sandeep, 2009 ▶). The flower are harvested from July to November and from May to December in North and South India (Sandeep, 2009 ▶). Various parts of the plant including stem, bark, leaves, flowers and roots are used for medicinal purposes (Arun et al., 2016 ▶).

**History of use**

It has been extensively used by Indian tribes as a popular remedy for different ailments including body and stomach pain and toothaches (Sandeep, 2009 ▶; Arun et al., 2016 ▶). It was also reported to have beneficial properties in treating amenorrhea, chronic constipation and dysmenorrhea (Sandeep, 2009 ▶; Arun et al., 2016 ▶). A decoction from leaves and roots was reported to be useful against headache and edema, as well as giddiness (Somanadhan et al., 1998 ▶). 

Various parts of *J. grandiflorum *are traditionally used in Indian medicine. For instance, jasmine leaves were applied to eliminate corns, fresh jasmine flowers and oil were used to cure sores and jasmine root extract was used to embrocate the eyes (Patnaik, 1993 ▶).

**Chemistry**

A number of studies detected secoiridoids, terpenoids, avonoids, tannins, saponins and flavonoids in various parts of the jasmine plant(Arun et al., 2016 ▶). Different compounds including phenolics, protocatechuic acid, triterpene and oleanolic acid were isolated from methanol extracts of the dried aerial parts of the jasmine plant (Sadhu et al., 2007 ▶). 

**Pharmacology**

*J. grandiflorum *was reported to possess various pharmacological activities including cytoprotective, anti-convulsant, anti-cancer (Sandeep, 2009 ▶; Arun et al., 2016 ▶) and dermal ulcers healing properties (Chaturvedi et al., 2013 ▶). Anethanolic leaves extract of *J. grandiflorum *was evaluated for its antiulcer, anti-oxidant, anti-nociceptive and anti-inflammatory activities while amethanolic extract was reported to have anti-inflammatory and anti-oxidant activities in both *in vitro* and *In vivo* models (Chaturvedi and Tripathi, 2011 ▶; Arun et al., 2016 ▶). 

*Invitro* enzymatic assays showed strong ACE inhibitory activity for the extracts obtained from the aerial parts of *J. grandiflorum *(Somanadhan et al., 1998 ▶; Arun et al., 2016 ▶; Patten et al., 2016 ▶). The ACE inhibitory activity of the aqueous, ethanol and acetone extracts of *J. grandiflorum* (whole plant) was 46, 60 and 78%, respectively (Somanadhanet al., 1999 ▶).

Arun and his colleagues reported the half maximal inhibitory concentration (IC_50_) values of jasmine to be 26-36μM (Arun et al., 2016 ▶). The IC_50_ values for ACE inhibition of secoiridoid aglycones of jasmine were 20-25µM (Kiss et al., 2008 ▶). Patten et al. reported relatively high ACE inhibitory activity )IC50 30μM) for Sambacein I-III isolated from *J. grandiflorum *(Patten et al., 2016 ▶). 

***Tribulu sterrestris*** **L.**

**Plant description and distribution**

*Tribulus *is a genus of 20 species belonging to Zygophyllaceae (Ukani et al., 1997 ▶). *T. terrestr is *commonly known as caltrop, is an annual species with opposite and pinnate leaves and yellow petals that grow up to 10-60cm in height (Ganzera Bedir et al., 2001 ▶; Chhatre Nesari et al., 2014 ▶). This plant is widely distributed in tropical, mild temperate areas and desert climates such as Asia, the Mediterranean region and Mexico (Dinchev Janda et al., 2008 ▶; Hussain et al., 2009 ▶; Martino-Andrade et al., 2010 ▶; Hashim et al., 2014 ▶). 

**History of use**

Ayurvedic medical documents record some characteristics of the plant including a sweet taste and its use to help digestion and cooling tempers. There is a document which shows that *T. terrestris was *used for bladder disorders and urinary stone (Chhatre Nesari et al., 2014 ▶).* T. terrestris *is used in Persian and Chinese folk medicine as a remedy for various disorders (Chhatre et al., 2014 ▶) including cough, polyuria, and dysuria and as a gastric stimulant and aphrodisiac (Ukani et al., 1997 ▶; Hussain et al., 2009 ▶).

Currently, *T. terrestris *is used as a food supplement (“Tribocard”) and in veterinary medicine to improve reproductive activity and fertilization. In addition, supplements containing *T. terrestis* are applied in cases of libido disorder (Evstatieva and Tchorbanov, 2011 ▶).

Various parts of the herb such as leaves, stem and roots are utilized as appetite suppressing and as astringents, cathartic, and anodyne (Ukani et al., 1997 ▶). 

**Chemistry**

Several phytochemical studies showed the presence of various chemical classes in *T. terrestris *(Ukani et al., 1997 ▶; Dinchev et al., 2008 ▶; Abirami and Rajendran, 2011 ▶). For example, saponins, flavonoids, alkaloids, cinammic acid amides and lignin amides were found in *T. terrestris*. Furostanol, spirostanol saponins and four sulphated saponins of tigogenin and diosgenin were also isolated from this plant (Ukani et al., 1997 ▶; Kostova and Dinchev, 2005 ▶; Hashim et al., 2014 ▶). The fruit and root of *T. terrestris* are rich in flavonoids, alkaloids, phytosteroids and glycosides (Ukani et al., 1997 ▶; Hashim et al., 2014 ▶) and its leaves contain diosgenin, gitogenin and chlorogenin (Hashim et al., 2014 ▶).

**Pharmacology**

*In vitro* data showed that the methanolic fraction of *T. terrestris* fruit extract decreases the level of reactive oxygen species (ROS) and protects against kidney cellular damage caused by mercuric chloride(Kavitha and Jagadeesan, 2006 ▶). It was shown that *T. terrestris* has anti-hypertensive effects mediated via inhibition of ACE especially in the kidneys (Tuncer et al., 2009 ▶). 

Sharifi et al. (2003b) ▶ in their evaluation of an aqueous extract of *T. terrestris *suggested that the BP lowering effect of the extract resulted from its ACE inhibitory activity (Sharifi et al., 2003 ▶). Anethnopharmacological investigation on Indian medical herbs reported ACE inhibitory activities for aqueous, ethanol and acetone extracts of *T. terrestris *(aerial parts). The inhibitory effect was dependent on the type of the extract with the aqueous extract having the highest ACEI activity (Somanadhan et al., 1999 ▶). 

***Vaccinium myrtillus*** **L.**

**Plant description and distribution**

*Vaccinium myrtillus*, commonly known as bilberry, is a medicinal plant belonging to the Ericaceae family (Matsunaga et al., 2009 ▶). It is also known as whortleberry, huckleberry, blueberry and European blueberry (Chu et al., 2011 ▶). *V. myrtillus *is a low-growing shrub with dark red or blue fruit that grows in forests, moors and mountainous regions of Asia, North America and Europe (Matsunaga et al., 2009 ▶; Persson et al., 2009 ▶; Song et al., 2010 ▶). 

**History of use**

Berries have a long history of medicinal use and it was also used in food and pharmaceutical products (Puupponen-Pimiä et al., 2008 ▶; Song et al., 2010 ▶). Wild berries are used in daily diet in Nordic regions (Puupponen-Pimiä et al., 2008 ▶). Also, extracts of the fruit are used as coloring agents in wine, jams and syrups (Ulbricht et al., 2009 ▶).

As a folk remedy, bilberry is used for treatment of ailments such as diarrhea (Puupponen-Pimiä et al., 2008 ▶), vascular disorders (Song et al., 2010 ▶), and mucus inflammation (Ulbricht et al., 2009 ▶). The dried fruit is also used for treatment of various eye disorders including eyestrain, and myopia and to promote night vision (Puupponen-Pimiä et al., 2008 ▶;Ulbricht et al., 2009 ▶). 

**Chemistry**

The berries contain high levels of phenolic acid, flavonoid, lignin and phenolic polymers (e.g. polymeric tannins) (Puupponen-Pimiä et al., 2008 ▶). Chemical studies on the bilberry fruit and its extracts showed the presence of water-soluble polyphenolic flavonoids especially anthocyanins that are considered to be responsible for the health-promoting properties of bilberry (Matsunaga et al., 2009 ▶; Ulbricht et al., 2009 ▶; Song et al., 2010 ▶; Chu et al., 2011 ▶). *V. myrtillus *contains different anthocyanins such as galactosides and glycosides of peonidin, petunidin, delphinidin, malvidin and cyaniding (Ichiyanagi et al., 2004 ▶; Persson et al., 2009 ▶; Lee et al., 2013 ▶).

**Pharmacology**

It was reported that the anthocyanins of bilberry have beneficial properties such as anti-oxidant (Matsunaga et al., 2009 ▶; Persson et al., 2009 ▶; Ulbricht et al., 2009 ▶), anti-platelet, the ability to facilitate collagen biosynthesis, vasoprotection (Matsunaga et al., 2009 ▶), anticancer and antibacterial effects (Persson et al., 2009 ▶). In addition, the bilberry was shown to possess anti-inflammatory, hypolipidemic and hypoglycemic effects (Ulbricht et al., 2009 ▶). Beside such properties, the bilberry was reported to have ocular and neuroprotective effects (Cravotto et al., 2010 ▶; Chu et al., 2011 ▶). 

In a randomized controlled clinical trial on hypertensive cases, an adverse correlation between a combination of polyphenols [green tea (100mg), grape seed (330mg), resveratrol (60mg), quercetin, ginkgo biloba and bilberry (60mg)] and diastolic blood pressure was observed. This observation may be related to the potential of the polyphenols in activation and production of NO (Biesinger et al., 2016 ▶). 

Persson et al. in their study on bilberry and its polyphenols, incubated the endothelial cells isolated from umbilical veins with bilberry 25E extract (containing the chloride salt of the anthocyanidins and myrtillin chloride) for 10 min. The results showed that *V. myrtillus* extract (0.0062, 0.0125, 0.025, 0.05 and 0.1mg/ml) could inhibit ACE activity in a dose-dependent manner. Proanthocyanidins (e.g. tannins) isolated from bilberry decreased fluid retention, inhibited the renin-angiotensin-aldosterone system and induced an anti-hypertensive effect (Persson et al., 2009 ▶). 

In a randomized placebo-controlled clinicaltrial on71 participants, two portions of berries were consumed daily by 35 participants for 8 weeks. Berry consumption reduced SBP by about 1.5mmHg (Cravotto et al., 2010 ▶).Moreover, treatment of spontaneously hypertensive stroke-prone rats with 3% blueberries for 2 weeks, decreased the level of ACE activity in the blood. However, it had no effect on ACE activity in the testis, lung, kidney or aorta (Wiseman et al., 2010 ▶).

***Vitis vinifera*** **L.**

**Plant description and distribution**

*Vitis *(grapevine) is a genus with around 80 species belonging to the Vitaceae family. *Vitis vinifera *is a species native to southwestern Asia, Central Europe and North America. These plants grow under subtropical and Mediterranean conditions (Terral et al., 2010 ▶). The history of domestication of grapevine dates back to the first millennium BC in a region between the black sea and Iran. Anatolia in the Asia part of Turkey has a notable role in the diversification of grape varieties (Terral et al., 2010 ▶; Yilancioglu and Cetiner, 2013 ▶). The wild grapevine is a heliophilous liana that possesses flaky bark and fruits – known as a grape- with green, red or purple (black) color (Terral et al., 2010 ▶). 

**History of use**

Consumption of grapes has a history of more than 6,000 years. Greek philosophers reported the healing properties of grape wine (Ismail et al., 2014 ▶). Consumption of grapes was mentioned in ‘‘the Canon of Medicine” by Avicenna as a natural remedy for oral disorders especially gingival health and loose teeth (Faridi et al., 2015 ▶). In Eber papyrus, there are documents describing the usage of grapes for urinary problems (Inoue and Craker, 2014 ▶). Other applications of grapes are treatment of diarrhea, hemorrhage and varicose vein (Ismail et al., 2014 ▶). Indeed, today, grape is cultivated for its fruit and juice worldwide (Terral et al., 2010 ▶).

**Chemistry**

Grape seeds are a rich source of proteins, high-value fatty oils, procyanidins and phenolic components (Zhou et al., 2011 ▶). The grape seeds also contains oxidative derivatives of catechin and epicatechin and viniferone A, B and C (Fan et al., 2004 ▶).The phenolic components found in all parts of grape are responsible for wine color, its bitter taste and astringency (Basha et al., 2004 ▶). Major phenolic components in the leaves are myricetin, ellagic acid, kaempferol, gallic acid and quercetin (Ismail et al., 2014 ▶). The leaves also contain a wide range of polyphenols such as flavonoids, anthocyanins and organic acids, mainly oxalic, malic and tartaric acid; fumaric, succinic acid are also found in trace amounts (Ismail et al., 2014 ▶). 

**Pharmacology**

Several studies suggested that* V. vinifera *has therapeutic applications such as reduction of ischemic/reperfusion damage (Bombardelli et al., 1997 ▶; Facino et al., 1999 ▶), anti-inflammation (Ismail et al., 2014 ▶), anti-oxidant (Bombardelli et al., 1997 ▶; Fauconneau et al., 1997 ▶; Pourghassem-Gargari et al., 2011 ▶), vasorelaxant (Gharib Naseri et al., 2010 ▶), bronchodilatory (Gharib Naseri and Heidari, 2006 ▶), hypolipidemic (Adisakwattana et al., 2010 ▶) and hypoglycemic effects (Ismail et al., 2014 ▶).

Procyanidins protect endothelial cells from peroxynitrite damage and induce relaxation in human arteries, suggesting their cardioprotective activity (Aldini et al., 2003 ▶). 

Various studies reported the anti-hypertensive effects of grape potentially through ACE inhibition (Godse et al., 2010 ▶; Borde et al., 2011 ▶; Afonso et al., 2013 ▶). The antihypertensive and antioxidant effects were observed after chronic administration of myricetin (100 and 300mg/kg, per oral, for 4 weeks) –an important flavonol of grapes - to deoxycorticosterone acetate -induced hypertensive rats. Following myricetin treatment using strips of ascending colon, the cumulative concentration-response curve of angiotensin II and serotonin shifted to right (Borde et al., 2011 ▶).

## Discussion

Medicinal plants and natural products have long been used for treatment of a broad range of diseases. Many Traditional Medical Systems including Persian Medicine have used herbal medicines to manage cardiovascular disorders (Sobhani et al., 2017 ▶). In the present article, we discussed medicinal plants with ACE inhibitory activities suggested by TPM, that are currently used in Iran for the treatment of BP. These plants also act through other BP lowering mechanisms. Garlic and its major compounds allicin and S-allylcystein were found to lower BP in human clinical trials and animal experiments with a number of mechanisms including inhibition of ACE, production of H_2_S, stimulation of NO and blockage of α adrenergic receptors and calcium channels. Cinnamon was reported to possess antihypertensive effects through inhibition of ACE, elevation of the endothelial NO and activation of the K-ATP channels in vascular smooth muscles. Jasmine and its compounds sambaceins I-III and *T. terrestris* also demonstrated remarkable ACE inhibitory activities. Bilberry and its proanthocyanidins could also serve as a potential antihypertensive medicine through ACE inhibition along with reducing fluid retention and blocking the renin-angiotensin-aldosterone system. Grapes also demonstrated BP lowering effects potentially via ACEI activities. All the extracts and their active compounds seem to act at relatively low concentrations which are theoretically applicable in human studies based on the principles of allometric scaling of experimental doses(Shakeri et al., 2016 ▶). Importantly, most of the mentioned plants and natural products demonstrated a wide spectrum of cardiovascular protective activities such as reducing blood glucose, inhibiting platelet aggregation, inhibition of cholesterol synthesis, antidiabetic effects, anti-inflammatory and antioxidant activities, reduction of ischemic/ reperfusion damage, etc. (Elkayam et al., 2001 ▶; Bombardelli et al., 1997 ▶; Facino et al., 1999 ▶; Sobhani et al., 2017 ▶). These activities along with BP lowering effects would potentially enhance the applicability of the plants.

Although a number of studies supported the traditional use of the discussed plants for the treatment of BP, the exact indications of use and doses are to be studied in future clinical trials. Fortunately, the mentioned plants are generally considered safe as they are mostly consumed as foods and food additives. However, further studies on potential adverse effects of such plants or herb-drug reactions are necessary. For instance, it is established that hydroxybenzoic and hydroxycinnamic acids which are present in many fruits including grapes can inhibit CYP3A4 activity in human liver microsomes by noncompetitive inhibition (Basheer and Kerem, 2015 ▶). Co-administration of garlic and warfarin was reported to increase international normalized ratio (INR) which leads to bleeding (Fugh-Berman, 2000 ▶). This effect is due to the presence of allicin which can interact with CYP3A4 (Rosenkranz et al., 2012 ▶). Accordingly, caution has to be given with respect to the co-administration of these plants with other prescribed medications.

Use of traditional remedies and medicinal plants in management of hypertension has become popularized in recent decades. The present article highlights the antihypertensive potential of six plants namely, garlic (*Allium sativum), *cinnamon (*Cinnamomum zeylanicum), *jasmine (*Jasminum grandiflorum), *caltrop (*Tribulus terrestris), *bilberry (*Vaccinium myrtillus)* and grape (*Vitis vinifera).*It is important to note that the differences in administered doses, manner of consumption, types of extracts, preparations and derivatives, personal habits, and other geographic and epidemiologic variables have an important role in the potential efficacy of these plants. The results of current and future animal and human studies can provide a better understanding of the mechanisms of actions by which extracts of these plants serve to ameliorate systolic and diastolic blood pressure. Moreover, further clinical trials are needed to evaluate the exact dosage of these plants and their active compounds, pharmacokinetic aspects, potential adverse effects and herb-drug interactions.
